# Sustainable Conversion of Biomass to Multiwalled Carbon Nanotubes and Carbon Nanochains

**DOI:** 10.3390/ma18051022

**Published:** 2025-02-26

**Authors:** Kevin R. McKenzie, Nathan A. Banek, Michael J. Wagner

**Affiliations:** Department of Chemistry, The George Washington University, Washington, DC 20052, USA; kevmckenzie@email.gwu.edu (K.R.M.J.); nbanek@email.gwu.edu (N.A.B.)

**Keywords:** biochar, valorization, carbon onions, nano-onions, laser pyrolysis, CNT, MWCNT, CNC

## Abstract

The conversion of biochar, the low value byproduct of pyrolysis bio-oil production from biomass multi-walled carbon nanotubes (MWCNTs) and carbon nanochains (CNCs), is reported. It is shown that biomass can be converted to long (>30 µm) carbon nanotubes with an anomalously deep (>280 nm) stacked-cup structure. A mechanism of the transformation that is consistent with previously reported graphitization of biochar, a “non-graphitizable” carbon, is proposed, suggesting the molten metal catalyst is absorbed into the biochar by capillary action, forming graphene walls as it percolates through pore structure. Graphite is formed when the diameter of the molten catalyst droplets is large (microns), while smaller droplets (submicron) form MWCNTs and still smaller (<100 nm) form CNCs. Branching in the biochar pore structure leads to subdivision of the catalyst droplets resulting in the progression from MWCNT to CNC formation. Very long MWCNTs (>50 µm) can be formed in the absence of CNCs by transforming lignite char rather than biochar, presumably due to the elimination of smaller branching pores during coalification. CNCs, in the absence of MWCNTs, can be formed in biochar by using low concentrations of catalyst nanoparticles formed by carbon thermal reduction of a metal salt during charring. The results presented suggest that developing methods to control the porosity of the char could yield the ability to rationally synthesize carbon nanotubes with control of length, breadth and wall thickness.

## 1. Introduction

Carbon nanotubes are one of the crystalline allotropes of carbon constructed from hexagonally bonded sp^2^ hybridized carbon atoms analogous to graphene sheets but in a hollow cylindrical form. CNTs can be single walled, known as single-walled carbon nanotubes (SWCNTs), or be composed of two or more walls, known as multi-walled carbon nanotubes (MWCNTs). While the diameter range of a CNT can be in the nanometer range, the length of a CNT may reach many microns. CNTs have high tensile strength, high elasticity, high electrical and thermal conductivity, low density and are chemically inert, properties that have made CNTs a promising nanomaterial group with applications ranging from quantum computing to drug delivery.

CNTs are synthesized by a variety of methods, including laser ablation, arc discharge and chemical vapor deposition (CVD). Laser ablation uses a pulsed laser to vaporize a graphite substrate in an electrical furnace to yield CNTs deposited on the cooler reactor surfaces at extremely high carbon purity levels and high (~70%) yield. This method produces primarily SWCNTs but is also the most expensive and lowest throughput method. Arc discharge uses extremely high temperatures, in excess of 3000 °C, to evaporate carbon atoms and form both SWCNTs and MWCNTs. Arc discharge has a maximum yield of only ~30% but can produce tubes with lengths of up to 50 microns [1].

The most widely used method of CNT production is CVD of carbon onto catalyst nanoparticles on a substrate. The substrate is heated to a high temperature, typically in excess of 700 °C, and exposed to a gas mixture composed of a process gas such as nitrogen or hydrogen and the carbon source, typically acetylene, ethylene or methane. The CNTs grow perpendicular to the substrate surface at the catalyst metal sites. The metal catalyst either remains at the nanotube base or moves with the tip of the nanotube, dependent on the adhesion between the catalyst and the substrate. CVD has the lowest cost and highest yield of current industrial production methods, but unfortunately, it is still an expensive process [2].

CNT synthesis from biomass promises to be both more environmentally friendly and less expensive than current industrial production. CNT production from a botanical precursor was first reported by Kumar and Ando in 2003 [3]. A variety of methods of CNT production from biomass have appeared since [4,5,6]. However, methods developed to date generally result in low-quality [5], short (>1 µm) MWCNTs [7,8,9,10,11]. While matching the quality of MWCNTs made by CVD has not been achieved yet, the results to date are promising.

It was recently reported that biochar and other non-graphitizable carbons could be converted to highly pure and crystalline graphite (biochar graphite or BCG) by a rapid, efficient and potentially inexpensive method [12,13,14], with a remarkable degree of agglomerate morphology control that allowed replication of commercial Li-ion battery graphite performance [14]. The synthesis of BCG has been shown to be accomplished by a mobile metal catalyst migrating through the pores of remarkably stationary biochar, allowing the rational control of the morphology of the resulting graphite [14]. This allows for the graphitization of porous carbons such as lignite and the failure to graphitize fusing bituminous coal due to its formation of chars with inaccessible pore structures [13]. This suggests that the BCG process cannot only convert biochar to flake or shaped graphite but also might be tuned to instead produce carbon nanotubes given the proper reaction conditions. Here we show that the suggestion is correct, providing an alternative, green route to multi-walled carbon nanotubes (MWCNTs) and/or the closely related carbon nanochains (CNCs). Furthermore, the reaction conditions, including catalyst and its loading, as well as the carbon feedstock, are investigated, with the results strongly suggesting a route to rational, selective synthesis of these structures with pre-determined dimensions.

## 2. Experimental

### 2.1. Materials

All materials were used as received unless otherwise noted. Hardwood sawdust (CrossRoad Sales LLC, Draper, UT, USA), lignite (mined in North Dakota and provided by North American Coal Inc., Plano, TX, USA) and <10 µm Fe metal (Alfa Aesar iron powder, 99.5%, Haverhill, MA, USA) were used as the starting materials.

### 2.2. Synthesis of MWCNTs

Biomass (sawdust, sieved to 100–140 mesh or 105–149 micron) or lignite with 1% by weight of Fe metal powder (Alfa Aesar, spherical, <10 micron, 99.9 + purity) were milled (80 mL steel cup, Fritsch GmbH, six 1 cm steel balls, 300 rpm, 30 min, Pulverisette 6, Fritsch GmbH, Idar-Oberstein, Germany). Pellets (20 mm) were formed and a centered hole (21/64”) drilled, followed by charring at 600 °C (30 min, 20 °C/min ramp) under inert gas (30 mL/min). The pellets were then placed on a steel rod (1/4”) and mounted vertically in a laser pyrolysis chamber. The pellets were subjected to laser pyrolysis (2 mm diameter spot, 200 W BWT Beijing laser, 980 nm) while rotating (one 48 s rotation).

Purification was conducted in a MARS 5 Digestion Microwave System (CEM Corp., Matthews, NC, USA, HNO_3_, 10 min ramp to 210 °C with 30 min hold). The room temperature mixture was diluted and the product recovered by vacuum filtration and further washing to neutralize.

### 2.3. Synthesis of CNCs

Biomass with FeCl_2_×4H_2_O salts were formed into pellets and charred as described in Section 2.2. Laser pyrolysis was conducted in an analogous fashion using a 10.4 µm wavelength laser (2 mm spot, 95% power, Firestar t60, Synrad Inc., Mukilteo, WA, USA). Purification was conducted as described in Section 2.2.

### 2.4. Activation of CNCs

Activation of the carbon nanochains was carried out by mixing the purified product with KOH in a 1:2 mass ratio and ball milled for 10 min. at 40Hz using a mini-mill (Pulverisette 23, Fritsch GmbH) with one ball (hardened stainless steel, 1.5 cm diameter). The powder was then heated under N_2_ or Ar gas (30 mL/min) in an alumina boat at 800 °C (20 °C/min ramp rate) for 10 min.

### 2.5. Characterization

Powder X-ray Diffraction (XRD). XRD diffractograms were collected from 20 to 60° 2θ (Rigaku Miniflex^+^). Igor Pro^®^ (WaveMetrics Inc., Portland, OR, USA) multi-peak fitting package was used for deconvolution of the diffractogram reflections with Voigt profiles.

Surface Area and Porosity Determination. BJH (Barret-Joyner-Halenda) pore size distributions were determined using from nitrogen adsorption isotherms (Tri-Star 3000, Micrometrics, Norcross, GA, USA). The saturation vapor pressure (P_0_) of N_2_ was measured at each temperature.

Scanning and Transmission Electron Microscopy (SEM and TEM). SEM micrographs were obtained at 1.00 and 2.00 kV accelerating voltage (in-lens secondary electron detector FEI Teneo LV, Hillsboro, OR, USA). TEM micrographs were captured at 200 kV (FEI Talos F200X TEM with a Ceta^TM^ 16M camera). TEM samples were deposited on 400 mesh Cu grids coated with 5–6 nm carbon film (Electron Microscopy Sciences, Hatfield, PA, USA).

## 3. Results and Discussion

### 3.1. Conversion of Biochar to Carbon Nanotubes

**Figure 1:**
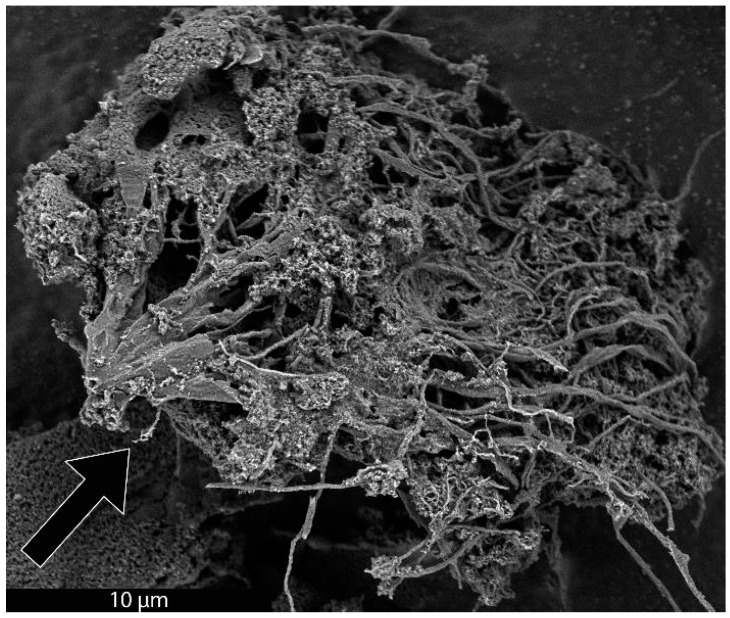
SEM image of a cluster of CNTs formed from biochar. The proposed entry area of Fe catalyst into a biochar particle is indicated with a black arrow.

**Figure 2:**
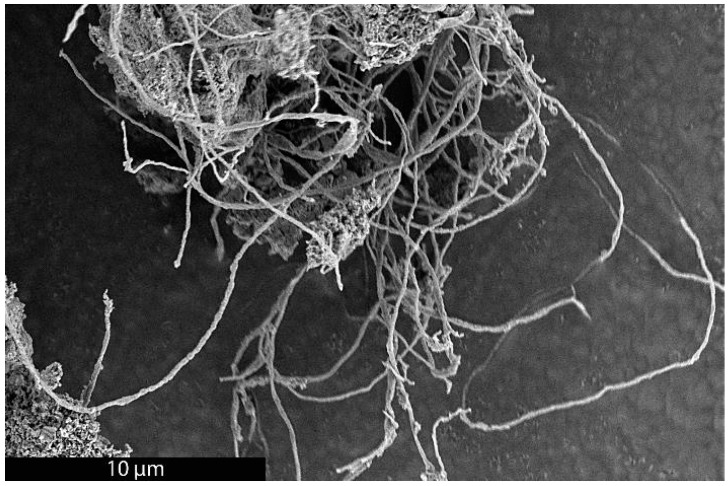
SEM image of a cluster of long CNTs made by the BCG process.

As previously mentioned, it was recently demonstrated that the synthesis of BCG is accomplished by a mobile metal catalyst migrating through stationary biochar, allowing the rational control of the morphology of the resulting graphite [14]. It was suggested that the molten catalyst enters the biochar through its network of pores, driven through the carbonaceous material by capillary action. Wicking of the molten catalyst into the biochar pores provides a synthetic opportunity; by keeping the catalyst loading low and the catalyst particle size small in comparison to that of the biochar, the catalyst can be confined to the pores, unable to absorb all of the carbonaceous precursors between the pores, only able to graphitize the pore walls. To investigate this, relatively large biomass particles (sawdust sieved to 100–140 mesh, 105–149 µm) were mixed with 1% *w*/*w* small (<10 µm) Fe, pressed into a pellet, charred at 600 °C, subjected to laser irradiation (200 W, single full 48 s rotation) and purified with HNO_3_. In addition, the experiment was repeated with ~80 µm Fe with all other conditions being identical. As expected, the sample with the larger Fe catalyst produced graphite. In contrast, the sample with the smaller Fe was apparently confined to the pore structure of the biochar, graphitizing the walls of the pores to produce carbon nanotubes (CNTs). As shown in Figure 1, the catalyst may have entered a biochar particle near the point indicated by the black arrow. A small number of rectangular graphite flakes appear at the proposed entry point, produced due to the high local catalyst concentration. From there, the molten metal proceeded to disperse, confined to the pores, transforming them into CNTs. In general, the nanotubes appear to be casted replicas of the pore structure of the biochar, with clusters composed of a mixture of thicker, long, relatively straight CNTs and interconnecting thinner, shorter, tortuous ones and carbon nanochains. A large proportion of the CNTs are very long (>30 µm), usually present in tangled clusters (Figure 2).

### 3.2. Mechanism of Carbon Nanotube Formation

**Figure 3:**
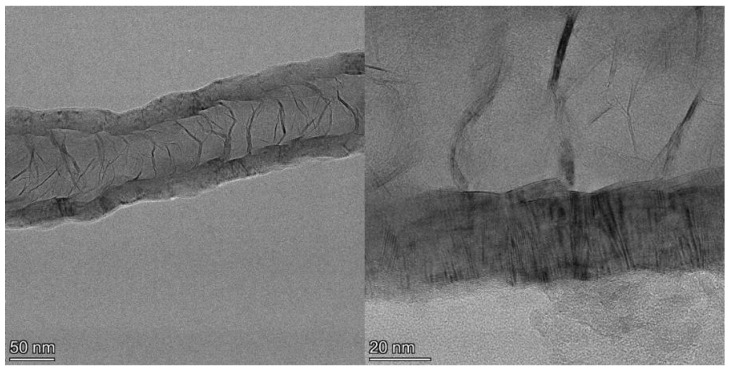
TEM image of section of a ~4 µm long CNT showing exterior and bridging walls (**left**) and higher resolution image (**right**) of the same tube showing wall lattice fringes and bridging walls joining the tube walls interior.

Examination of the long nanotubes with HRTEM finds their exterior diameters to be ~100–130 nm, the walls to be ~25–30 nm thick and the intershell spacing to be ~3.40 Å (Figure 3). Periodically, graphene layers bridge the interior of the tube to join the walls, forming new interior walls. The bridging graphene walls occur at a somewhat regular interval but vary in thickness from ~2–10 nm, with the vast majority being on the very low end of that range.

Despite the addition of interior walls, the overall thickness of the tube walls remains relatively constant, suggesting that the walls that are added from the interior eventually terminate at the exterior. Examination of images of the entire cross section of longer segments of the tubes shows that the vast majority of the walls originating in the tube interior are continuous across the tube, intersecting with and joining the exterior walls on both sides, indicative of a stacked-cup structure of nested, capped, truncated cones. However, the cups are unusually long, with each cone forming part of the CNT walls for >280 nm after joining from the interior, or approximately six times the field of view of the HRTEM image shown in Figure 3 (left). The angle of the graphene cups in the walls with respect to the tube principal axis is very small, ~2°, giving the tube walls the appearance of being continuous until close inspection.

**Figure 4:**
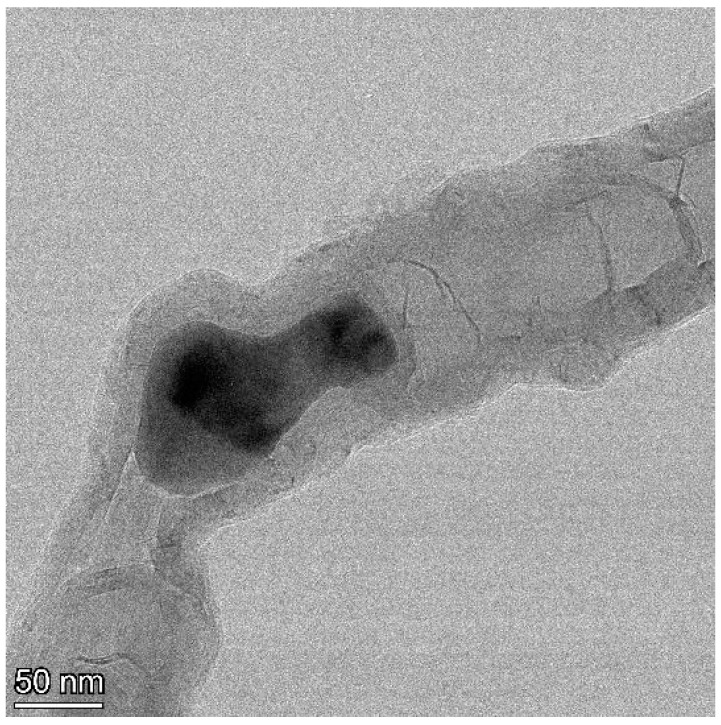
TEM image of Fe catalyst particle encapsulated in a MWCNT.

TEM observations of the CNTs following purification show that nearly all of the Fe catalyst has been removed, despite the seemingly compartmentalized structure of the tubes. Extensive TEM observation yielded one example of a Fe catalyst particle that was not removed during purification (Figure 4). The particle is located at a sharp bend, a “knee”, in the tube, giving the appearance of cojoined tubes. It seems highly unlikely that the catalyst particle created the tubes on both sides of the knee by translating in back and forth along their lengths. The bridging wall curvature is the same on either side, consistent with unidirectional movement. In addition, bidirectional movement would require that the bridging walls be open (passable), which should have allowed the catalyst removal during purification. Alternatively, the segment in the upper right could have terminated at the end of another already formed tube; however, the joining wall structure appears continuous, albeit somewhat disrupted. In addition, the interior diameter of the tube to the right (~69 nm) is considerably larger than that to the left of the catalyst (~47 nm), consistent with the molten Fe splitting into a portion left behind as seen in the image and a smaller portion that moved ahead, creating a smaller diameter tube.

The morphology of the stacked-cup CNTs reported here is similar to those made by gas-phase CVD methods, albeit with unusually long continuous graphene cones and a very small angle between the graphene cones in the walls and the tube principal axis [15,16]. However, CVD growth relies on the decomposition of gaseous carbon precursors that are transported to the catalyst, while here the carbonaceous precursor, biochar, is largely immobile and likely not decomposed to species smaller than large aromatic graphene-like molecules. Thus, the BCG method is more analogous to a previously reported arc discharge synthesis method, producing tubes from a mixture of Fe and coal [17]. However, the tubes made by arc discharge had a distinctive bamboo-like structure, a chain of individual <100 nm compartments with each terminating at the start of the next, rather than the stacked-cup structure reported here.

It is likely that the formation of the stacked-cup CNTs reported here is in most respects similar to their formation by vapor transport methods [18]. Following entry of molten Fe catalyst in the pores of the biochar, conjugated carbonaceous molecular fragments are released from the biochar by breaking of their crosslinking bonds, either due to laser irradiation or interaction with the Fe, and absorbed by the Fe. Graphene walls on the back and sides of the catalyst are formed, with repeated lengthening and contraction of the molten catalyst preventing wall formation in the front and giving access to additional carbonaceous building blocks. This process differs from CVD methods in the chemical nature of these building blocks and that the catalyst percolates through the branching pore structure of the char, resulting in network of large and small CNTs, rather than uniform growth of individual CNTs.

### 3.3. Conversion of Biochar to Carbon Nanochains

**Figure 5:**
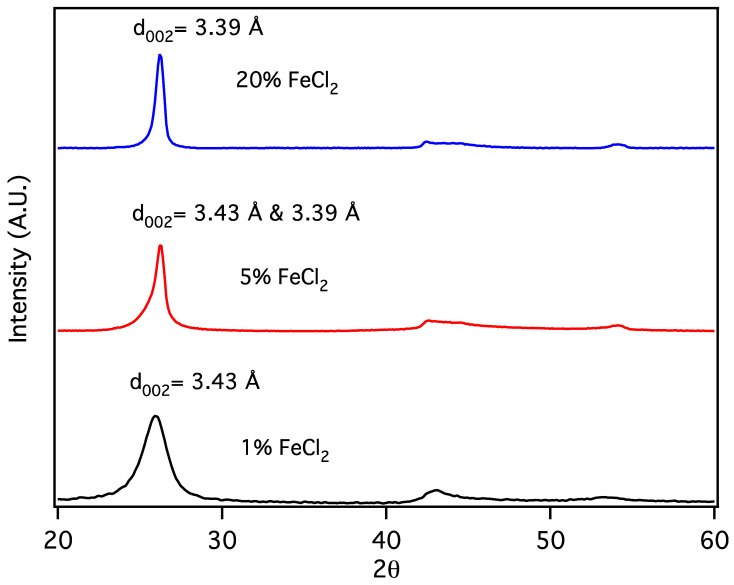
X-Ray diffractograms of carbon nanostructures made with 20, 5 and 1% *w*/*w* FeCl_2_ catalyst.

Carbon nanochains (CNCs) are produced from biochar as a minority species in conjunction with CNTs, appearing to be formed in smaller pores or when, due to division at pore branches, the volume of molten Fe catalyst droplet is reduced below some critical value. The initial volume of the Fe catalyst particles can be further reduced by forming them from a metal salt, eliminating the production of CNTs in favor of CNCs. Sawdust was mixed with 1, 5 and 20% *w*/*w* FeCl_2_ × 4H_2_O and subjected to thermal pyrolysis, forming Fe nanoparticles embedded in biochar [19], followed by laser pyrolysis. Following purification, powder X-Ray diffraction patterns (Figure 5) for the resulting products are similar to that expected for graphite, with a reflection due to diffraction from stacked carbon layers, commonly referred to as the “d_002_” reflection, despite its crystallographic designation only being proper for the graphite structure. Here, the reflection at ~26° 2Θ corresponds to the graphene interlayer spacing of the multilayer graphene shells of the multilayer graphene nanospheres (MGNSs) and the CNCs.

**Figure 6:**
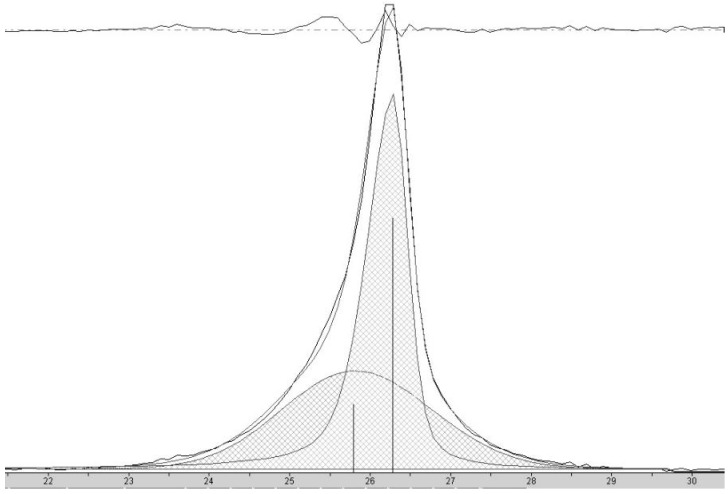
X-ray diffractogram of carbon nanostructures made with 5 wt% FeCl_2_ catalyst showing the peak corresponding to the interlayer spacing of the graphene walls of nanostructures. Deconvolution of the data shows the presence of two reflections (shaded), the sharper peak with its maximum (indicated by the vertical line to zero) at higher angle corresponding to HCNS and the broader peak with its maximum at lower angle to CNC. The sum of the fit peaks and the data are overlayed and the difference between the sum of the deconvoluted peaks and the data is shown at the top of the plot, demonstrating a good fit.

**Figure 7:**
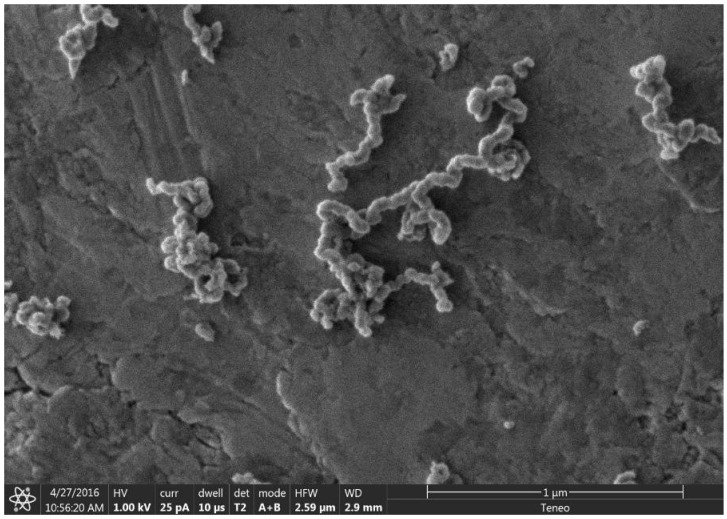
SEM image showing the chain nature of the carbon nanochains on a carbon tape coated SEM stub.

**Figure 8:**
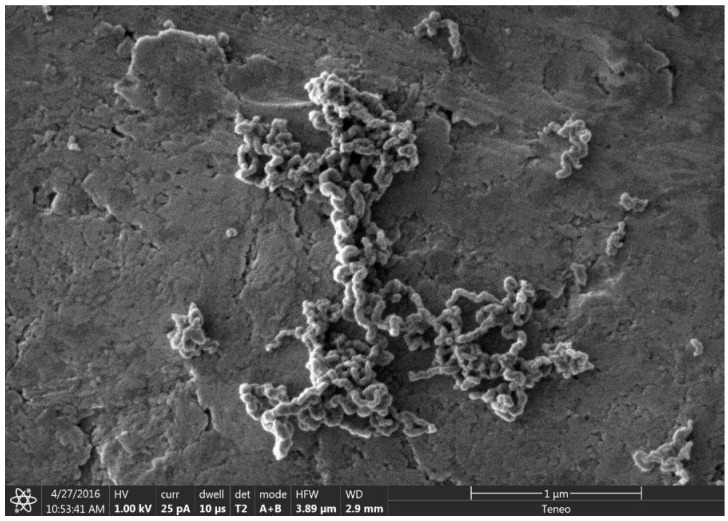
SEM image showing the chain nature of the CNCs curled up into masses on a carbon tape coated SEM stub.

At high concentrations of metal salt (e.g., 20% *w*/*w*), a single d_002_ reflection is present corresponding to the interlayer spacing of the graphene multilayers wall of multilayered graphene nanoshells [19]. When the concentration is decreased to 5% *w*/*w*, the reflection asymmetrically broadens. Deconvolution of the data (Figure 6) shows the presence of two reflections (shaded), the sharper reflection with its maximum (indicated by the vertical line to zero) at higher angle corresponding to the interlayer spacing of MGNSs (3.39 Å) and the broader reflection with its maximum at lower angle to CNCs (3.43 Å). The sum of the fits and the data are overlayed, and the difference between the sum of the deconvoluted reflections and the data is shown at the top of the plot, demonstrating a good fit, consistent with the product consisting of a mixture of MGNSs and CNCs. At low concentration (e.g., 1%), the product is essentially only CNCs, as can be seen from the absence of the XRD reflection corresponding to MGNSs (Figure 5). SEM images of the CNCs show the twisting, chain-like nature of capsule shaped links, generally several microns in length (Figure 7). However, most of the CNCs are curled up into irregular masses (Figure 8).

**Figure 9:**
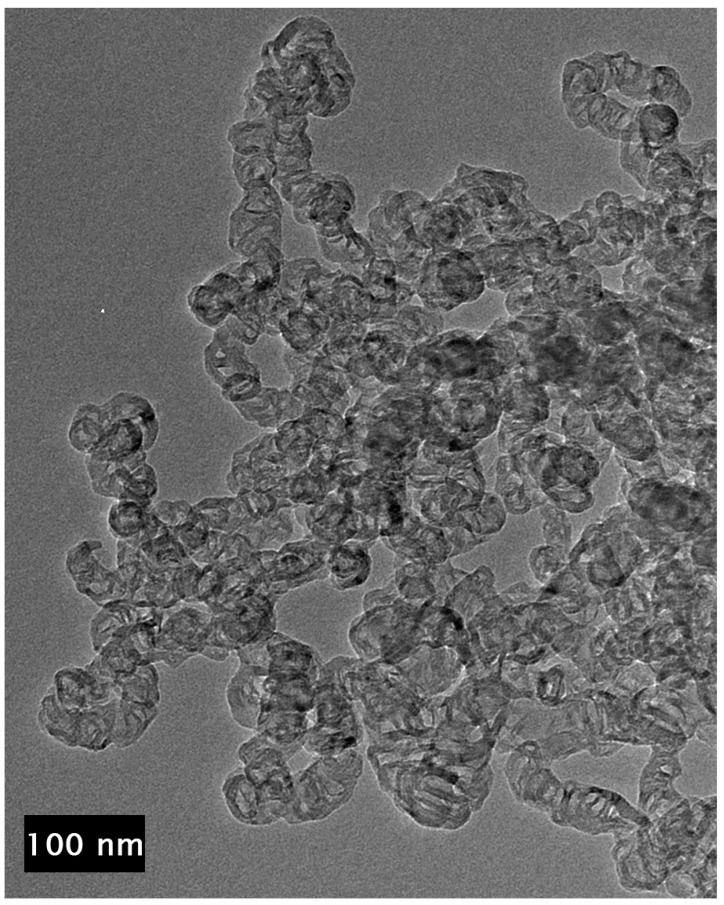
TEM image of the unpurified CNCs showing no metal inside the individual links of the chains.

**Figure 10:**
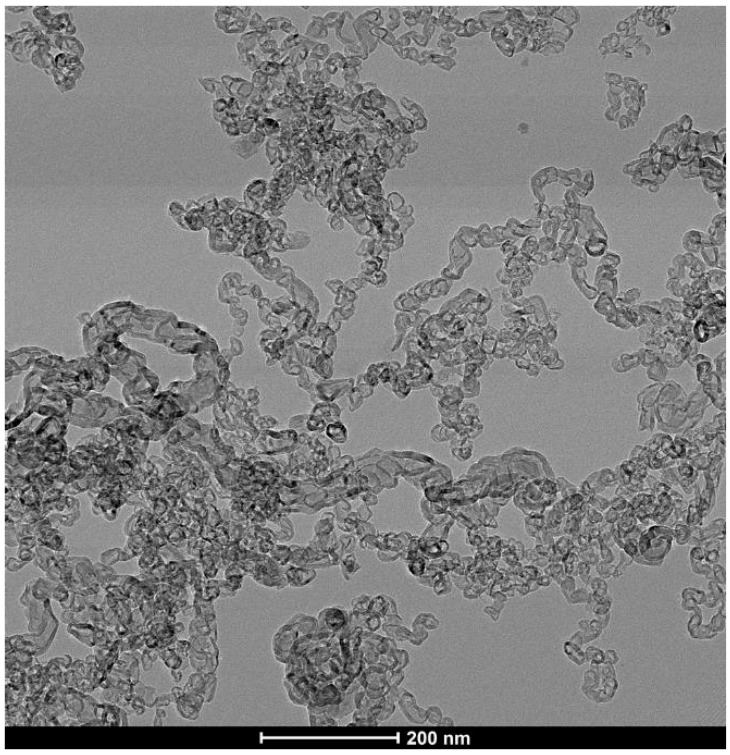
TEM image of purified CNCs showing that the chains consist of connected carbon shell links.

TEM images of the unpurified CNCs show that they consist of chains of hollow multilayer carbon shells (Figure 9). The vast majority of the interiors of the links are devoid of metal catalyst. This is in contrast to MGNS where each nanosphere forms around a metal particle, enveloping the metal until it is leached out during purification [19]. A small amount of material that appears to be amorphous carbon can be observed in some places on the surface of the CNC. This material is removed by purification, allowing for more ready observation of the CNC structure (Figure 10). The links that compose the chains are irregular in shape, varying from spheroidal to capsular. The internal structure appears to be stacked-cup, akin to the CNTs reported above, but with wall lengths that are as much as an order of magnitude less, ranging from ~20 to 100 nm, with most being less than 50 nm. This suggests a similar formation mechanism, only with smaller molten Fe masses percolating through the biomass. Chain width is generally ~15–30 nm, but as large as ~50 nm in places.

**Figure 11:**
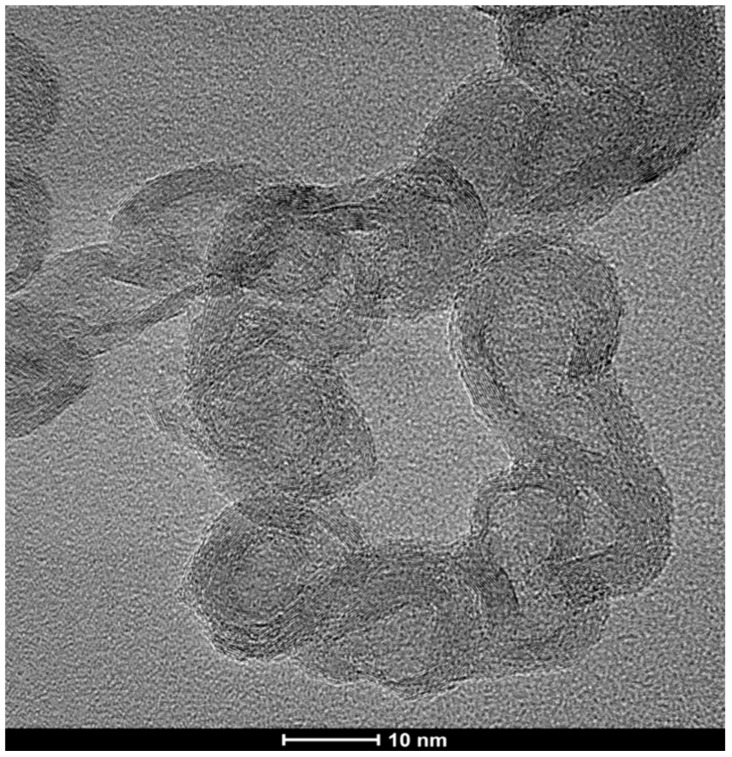
High resolution TEM image of carbon nanochains showing that the walls of the links in the chains are interconnected graphene multilayers.

**Figure 12:**
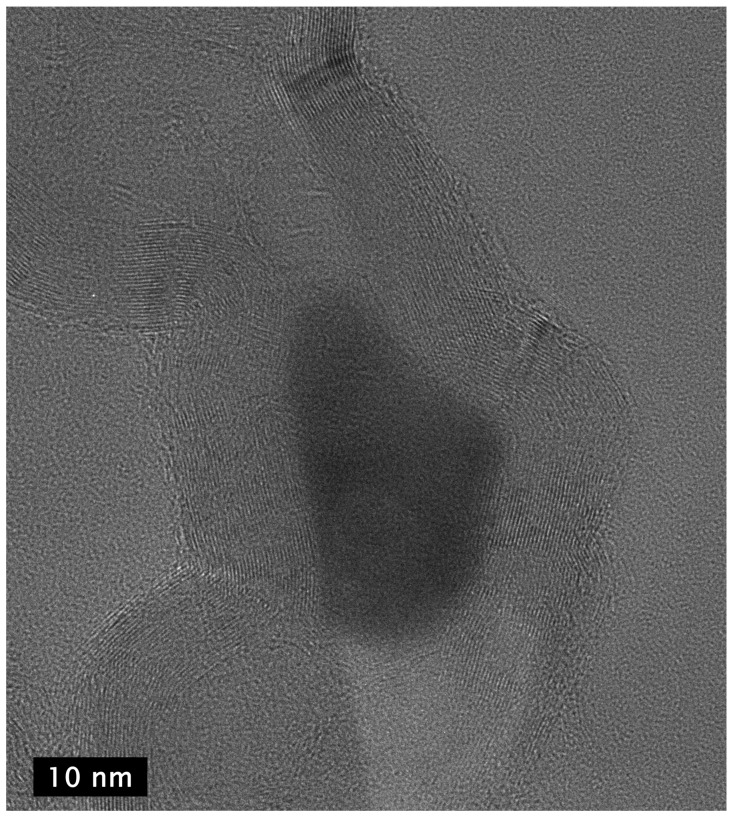
HR-TEM image of two adjacent links of a carbon nanochain showing that the walls of the links in the chains are interconnected graphene multilayers and the presence of metal catalyst (dark area in center) that presumably had been expelled from one link to form an adjacent link before solidifying as it cooled, terminating the growth of the chain at the link in which it is seen in the image.

HRTEM images of the CNCs further demonstrate the stacked-cup nature of their wall structure (Figure 11). The walls, generally ~3–4 nm in width (9–12 layers) but as wide as 10 nm in places, are shared by adjacent links, with many spanning multiple links. The width of the enclosed interior region is generally ~5–8 nm at their widest spans, but some are found to be modestly smaller and can exceed 25 nm in places. The purification procedure removes the metal catalyst, but in very rare cases, a small amount of the catalyst remains encapsulated (Figure 12).

**Figure 13:**
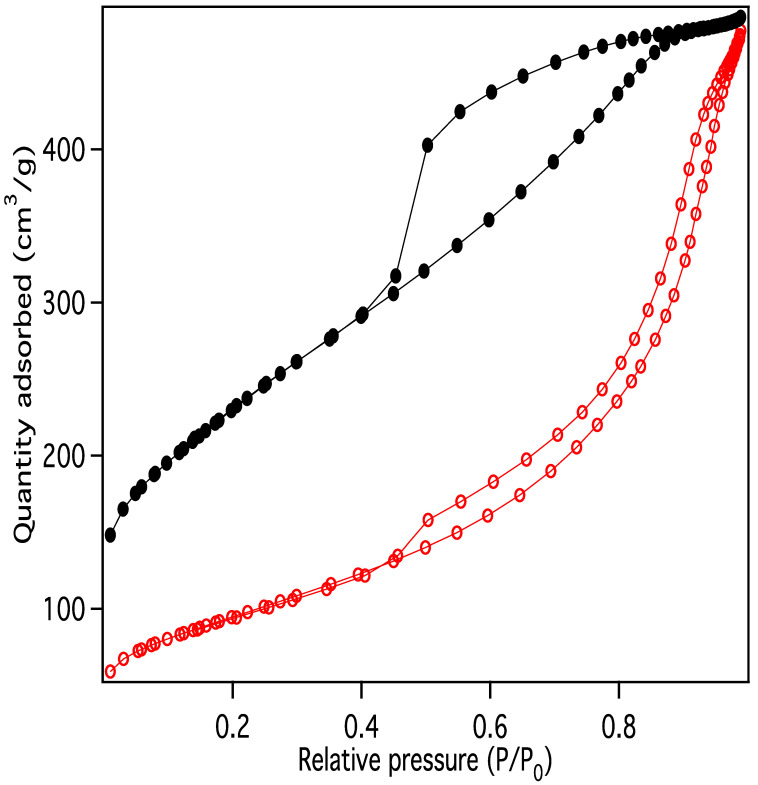
Nitrogen adsorption/desorption data for purified carbon nanochains before (red) and after (black) activation.

**Figure 14:**
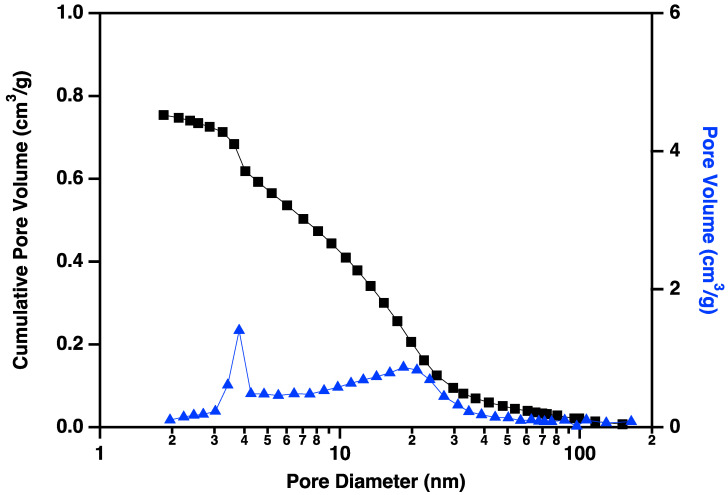
Cumulative pore volume (black squares linked by lines to guide the eye) and pore volume (blue triangles linked by lines to guide the eye) plotted against pore diameter for carbon nanochains that have been purified according to the procedure described above but not activated. Note the small volume of small (~4 nm) pores prior to the activation process.

**Figure 15:**
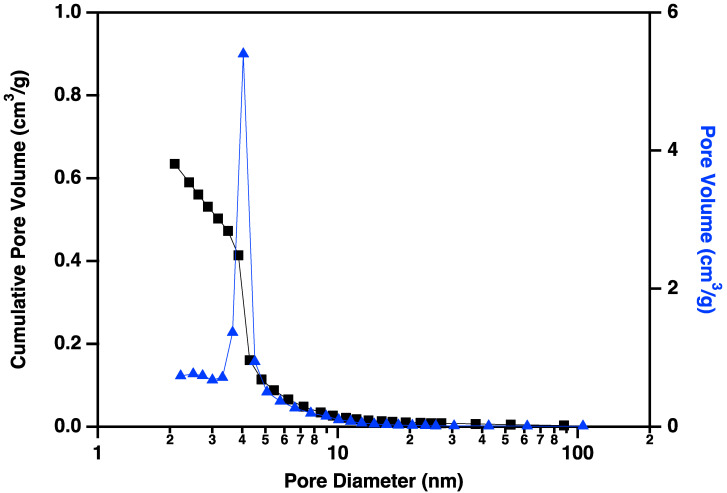
Cumulative pore volume (black squares linked by lines to guide the eye) and pore volume (blue triangles linked by lines to guide the eye) plotted against pore diameter for carbon nanochains that have been purified and activated according to the procedure described above. Note the large volume of small (~4 nm) pores that have been made accessible by the activation process.

Nitrogen adsorption/desorption display IUPAC Type IV behavior, typical of capillary condensation in mesoporous materials (Figure 13) [20]. The isotherm adsorption curve increases with gradually greater slope as pressure is increased. The desorption curve is very similar in shape and slope, but hysteretic, displaying behavior that is typical of “wedge-shaped pores”, those formed at the intersection of abutting materials. BJH (Barret-Joyner-Halenda) pore size distributions show a peak in the pore diameter at ~4 nm, closely matching the diameters of the interiors of the majority of the CNC links. This indicates that the CNC walls have openings that allow access to the interiors of some of the links (Figure 14), in addition to the primary porosity provided by the confluence of neighboring CNC chains. Activation of the CNCs transforms the isotherms to the hysteretic shape typical of “ink bottle shaped” pores, and the change in porosity is dominated by pores of ~4 nm (Figure 15), as would be expected if the additional porosity were due to increased access to the interiors of the links (Figure 13).

### 3.4. Conversion of Lignite to Carbon Nanotubes

**Figure 16:**
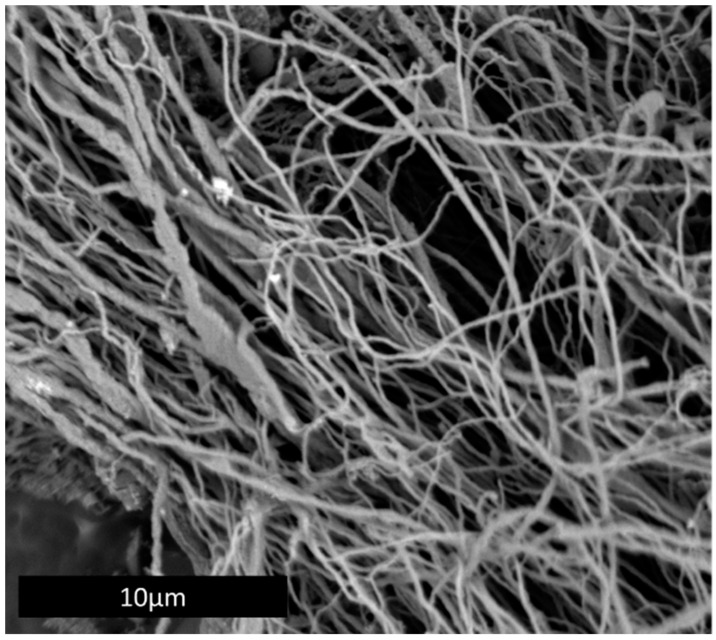
MWCNTs formed from lignite.

Formation of CNTs from biomass as described above results in CNCs as a minority co-product. Presumably this is due to branching in the biochar pores, reducing the size of the molten Fe catalyst as it divides to fill multiple pores at these junctions. In contrast, CNC formation is greatly reduced or eliminated when the CNTs are formed from lignite by the same method (Figure 16), resulting in large clusters of CNTs. The CNTs formed from lignite are generally longer and straighter than those formed from biomass, with a very large proportion >50 µm with diameters from 100 nm to 400 nm. The absence of CNC formation from lignite is probably due to the loss of the finer micropore structure during the coalification process. When lignite is charred, it fractures and forms straight and ordered micropores [21] without the smaller pores found in biochar, resulting in the synthesis ordered bundles of long CNTs free of CNCs.

## 4. Conclusions

Biomass and lignite can be converted to long carbon nanotubes and/or carbon nanochains and lignite by a relatively simple, rapid laser pyrolysis in the presence of iron catalysts. While the tubes presented here display a stacked-cup structure, their walls are notably long, as are their overall lengths. Their wall thickness is probably a function of the size of the metal catalyst from which they form, a parameter that may be tunable. Furthermore, their size and length appear to be determined by the pore structure of the biomass char, giving rise to the possibility of engineering templates for tubes of desirable dimensions. However, it should be noted that this will require the development of methods to rationally modify the pore structure of biochar. The wall structure may be a function of reaction parameters, particularly growth temperature, as was found with conventional nanotube production, giving rise to the possibility that with modest modifications, “infinite” wall carbon nanotubes may be accessible. In addition, the ecological advantages of the BCG method carry over to nanotube synthesis, as does the low cost, potentially dramatically reducing the high cost of nanotube production. CNCs are potentially less expensive still and, while similar to MWCNTs, are sufficiently different to merit investigation. Taken as a whole, the results here suggest that the size, shape, structure and morphology of MWCNTs and CNCs could be rationally selected by a combination of reaction conditions and control of biochar porosity.

## Data Availability

The original contributions presented in the study are included in the article, further inquiries can be directed to the corresponding author.

## References

[1] Inagaki M., Kang F., Toyoda M., Konno H. (2013). Advanced Materials Science and Engineering of Carbon.

[2] Pant M., Singh R., Negi P., Tiwari K., Singh Y. (2021). A comprehensive review on carbon nano-tube synthesis using chemical vapor deposition. Mater. Today Proc..

[3] Kumar M., Ando Y. (2003). Camphor–A botanical precursor producing garden of carbon nanotubes. Diam. Relat. Mater..

[4] Vivekanandhan S., Schreiber M., Muthuramkumar S., Misra M., Mohanty A.K. (2017). Carbon nanotubes from renewable feedstocks: A move toward sustainable nanofabrication. J. Appl. Polym. Sci..

[5] Janas D. (2020). From Bio to Nano: A Review of Sustainable Methods of Synthesis of Carbon Nanotubes. Sustainability.

[6] Ge L., Zuo M., Wang Y., Wang R., Rong N., Qi Z., Zhao C., Zhang Y., Xu C. (2024). A review of comprehensive utilization of biomass to synthesize carbon nanotubes: From chemical vapor deposition to microwave pyrolysis. J. Anal. Appl. Pyrolysis.

[7] Osman A.I., Farrell C., Al-Muhtaseb A.a.H., Harrison J., Rooney D.W. (2020). The production and application of carbon nanomaterials from high alkali silicate herbaceous biomass. Sci. Rep..

[8] Qu J., Luo C., Cong Q., Yuan X. (2011). Carbon nanotubes and Cu–Zn nanoparticles synthesis using hyperaccumulator plants. Environ. Chem. Lett..

[9] Dubrovina L., Naboka O., Ogenko V., Gatenholm P., Enoksson P. (2013). One-Pot synthesis of carbon nanotubes from renewable resource: Cellulose acetate. J. Mater. Sci..

[10] Kang Z., Wang E., Mao B., Su Z., Chen L., Xu L. (2005). Obtaining carbon nanotubes from grass. Nanotechnology.

[11] Hidalgo P., Navia R., Hunter R., Coronado G., Gonzalez M. (2019). Synthesis of carbon nanotubes using biochar as precursor material under microwave irradiation. J. Environ. Manag..

[12] Banek N.A., Abele D.T., McKenzie K.R., Wagner M.J. (2018). Sustainable Conversion of Lignocellulose to High-Purity, Highly Crystalline Flake Potato Graphite. ACS Sustain. Chem. Eng..

[13] Wagner M.J., Banek N.A., Abele D.T., Mckenzie K.R. (2022). Method and Systems for the Production of Crystalline Flake Graphite from Biomass or Other Carbonaceous Materials. U.S. Patent.

[14] Banek N.A., McKenzie K.R., Abele D.T., Wagner M.J. (2022). Sustainable conversion of biomass to rationally designed lithium-ion battery graphite. Sci. Rep..

[15] Jia Z., Kou K., Qin M., Wu H., Puleo F., Liotta L.F. (2017). Controllable and Large-Scale Synthesis of Carbon Nanostructures: A Review on Bamboo-Like Nanotubes. Catalysts.

[16] Feng L., Xie N., Zhong J. (2014). Carbon Nanofibers and Their Composites: A Review of Synthesizing, Properties and Applications. Materials.

[17] Li Y.F., Qiu J.S., Zhao Z.B., Wang T.H., Wang Y.P., Li W. (2002). Bamboo-Shaped carbon tubes from coal. Chem. Phys. Lett..

[18] Kumar M., Yellampalli S. (2011). Carbon Nanotube Synthesis and Growth Mechanism. Carbon Nanotubes-Synthesis, Characterization, Applications.

[19] Mckinnon T.J., Herring A.M., McCloskey B.D. (2005). Laser Pyrolysis Method for Producing Carbon Nano-Spheres. U.S. Patent.

[20] Sing K.S.W. (1985). Reporting physisorption data for gas/solid systems with special reference to the determination of surface area and porosity (Recommendations 1984). Pure Appl. Chem..

[21] Song Y., Xie J., Xin L., Fu H. (2019). SEM Image Analysis of Pore and Fracture Characteristics of Lignite Under Temperature Gradient. Geotech. Geol. Eng..

